# ‘It depends on where you were born…here in the North East, there’s not really many job opportunities compared to in the South’: young people’s perspectives on a North-South health divide and its drivers in England, UK

**DOI:** 10.1186/s12889-024-19537-z

**Published:** 2024-07-29

**Authors:** Hannah Fairbrother, Nicholas Woodrow, Eleanor Holding, Mary Crowder, Naomi Griffin, Vanessa Er, Caroline Dodd-Reynolds, Matt Egan, Steph Scott, Carolyn Summerbell, Emma Rigby, Philippa Kyle, Nicky Knights, Helen Quirk, Elizabeth Goyder

**Affiliations:** 1 Faculty of Health, University of Sheffield, Sheffield, S10 2LA UK; 2 SCHARR, University of Sheffield, Sheffield, S1 4DA UK; 3 Population Health Sciences Institute, Faculty of Medical Sciences, Fuse, Newcastle University, Newcastle upon Tyne, NE1 4LP UK; 4 Department of Health Services Research and Policy, London School of Hygiene and Tropical Medicine, London, WC1E 7HT UK; 5 Department of Sport and Exercise Sciences, Fuse, Durham University, Durham, DH1 3LA UK; 6 Association for Young People’s Health, London, SE1 4YR UK; 7 Nicky Knights, Social Sciences, University of Westminster, London, W1W 6UW UK

**Keywords:** Health inequalities, North-South divide, Social determinants of health, Young people, Qualitative

## Abstract

**Background:**

Improving the public’s understanding of how regional and socioeconomic inequalities create and perpetuate inequalities in health, is argued to be necessary for building support for policies geared towards creating a more equal society. However, research exploring public perceptions of health inequalities, and how they are generated, is limited. This is particularly so for young people. Our study sought to explore young people’s lived experiences and understandings of health inequalities.

**Methods:**

We carried out focus group discussions (*n* = 18) with 42 young people, aged 13–21, recruited from six youth organisations in England in 2021. The organisations were located in areas of high deprivation in South Yorkshire, the North East and London. Young people from each organisation took part in three interlinked focus group discussions designed to explore their (i) perceptions of factors impacting their health in their local area, (ii) understandings of health inequalities and (iii) priorities for change. Due to the Covid-19 pandemic, most discussions took place online (*n* = 15). However, with one group in the North East, we carried out discussions face-to-face (*n* = 3). Data were analysed thematically and we used NVivo-12 software to facilitate data management.

**Results:**

Young people from all groups demonstrated an awareness of a North-South divide in England, UK. They described how disparities in local economies and employment landscapes between the North and the South led to tangible differences in everyday living and working conditions. They clearly articulated how these differences ultimately led to inequalities in people’s health and wellbeing, such as linking poverty and employment precarity to chronic stress. Young people did not believe these inequalities were inevitable. They described the Conservative government as prioritising the South and thus perpetuating inequalities through uneven investment.

**Conclusions:**

Our study affords important insights into young people’s perceptions of how wider determinants can help explain the North-South health divide in England. It demonstrates young people’s contextualised understandings of the interplay between spatial, social and health inequalities. Our findings support calls for pro-equity policies to address the structural causes of regional divides in health. Further research, engaging young people in deliberative policy analysis, could build on this work.

**Supplementary Information:**

The online version contains supplementary material available at 10.1186/s12889-024-19537-z.

## Introduction

Inequalities in health are evident both between and within high and low and middle -income countries [1–4]. However, England has especially stark regional variations in health, particularly between the North and South of the country [5]. While there is no clear definition of a North-South divide in England (and popular discourse regarding a North-South divide may not adhere to strict geographical boundaries), there are acknowledged and well-established social, cultural and economic disparities, typically drawn between London and the South-East of England, and the rest of the country [6, 7]. Recent analysis by the Institute for Public Policy Research highlights ‘continued and growing regional divides in productivity, incomes, job creation, unemployment, pollution, emissions and educational outcomes’ [6 p.5] and the authors warn that ‘the North is too often at the sharp end of these inequalities’ [6 p.5]. Young people are acutely impacted by such regional disadvantages, with the North East having a higher proportion of young people not in employment, education or training than London and the South East [8].

Geographically patterned socioeconomic inequalities are inextricably linked to health. In England, people in the North live shorter, sicker lives than people in the South [9–11]. Health inequalities are evident across the lifecourse, with infant mortality rates consistently higher in the North, and life expectancy in the North East averaging three years lower than in London and the South East [8, 12]. Further, the three Northern regions (North East, North West and Yorkshire and The Humber) have ‘the highest rates of people reporting ‘bad’ or ‘very bad’ health’ [9 p.5]. Taylor-Robinson et al. (2021) underscore that at the heart of the North-South health divide lie ‘differences in exposure to poverty and the resources needed for health; differences in exposure to health-damaging environments; and differences in opportunities to enjoy protective conditions’ [13 p.14]. The North-South health divide is therefore a striking example of intermeshed geographic and socioeconomic inequalities.

Consistent with the evidence, there is widespread agreement amongst the academic community that tackling health inequalities requires change in the unequal distribution of key social determinants of health (for example, housing, employment, education and wealth) [14]. However, this has not been reflected in national policy and in recent years health inequalities in England have widened, including growing disparities between the North and South of England [9, 10]. The government elected in 2010 implemented a series of austerity measures designed to reduce the role of the state and ‘recover’ from the global recession [15]. Changes to the tax and benefit system, cuts to health and social care and local authority budgets, and wage stagnation (coupled with the fallout of the UK’s exit from the European Union) have all contributed to rising regional inequalities [16]. Further, the Covid-19 pandemic has exacerbated existing inequalities, with those lower down the socioeconomic ladder being disproportionately affected both in economic and health terms [17, 18]. The North of England has fared particularly badly [10]. Despite rhetoric of ‘Build Back Fairer’ and ‘Levelling Up’ following the pandemic in the UK [19, 20], the ongoing Cost-of-Living crisis looks set to further cement and exacerbate socioeconomic and regional inequalities in health. Low-income households are disproportionately affected by the rising costs of living [21], and charitable organisations providing frontline support to underserved communities are ‘running on empty’ [16].

In sum, there is a wealth of quantitative, epidemiological evidence highlighting deep-rooted and long-term socioeconomically and regionally patterned health inequalities in England today [9] and evidence that the North-South health divide is growing [22, 23]. In contrast, however, we know relatively little about how people across England, particularly young people, experience and perceive health inequalities in the context of their everyday lives [24] and their ideas about potential policy responses. Understanding England’s stark socioeconomic and regional inequalities from the perspectives of the communities experiencing them is vital if we are to develop policies to promote greater health equity [25]. As Popay et al., (1998) argue: ‘Attention to the meanings people attach to their experiences of places and how this shapes social action could provide a missing link in understanding the causes of inequalities in health’ [26 p.639]. Similarly, Kapilashrami et al. (2015) argue that an ‘ongoing process of dialogue through community mobilising, action research, movement building and public health advocacy’ is necessary to develop ‘targeted policy proposals for improvements in population health’ [27 p.416]. Further, a recent study by McHugh and colleagues (2023) highlights that policy actors perceive public participation as instrumental in policy development in two overlapping ways [28]: as evidence to improve policies to tackle health inequalities, and as key to achieving public acceptance for implementing more transformative policies. Young people have been disproportionately impacted by rapid changes in employment opportunities and labour markets, disruption to education and educational transitions and trends in worsening mental health, and we now have a generation of young people experiencing considerable intergenerational inequalities [29]. Young people’s experiences of and perspectives on health inequalities are therefore a critical part of the public voice required by policymakers [29].

### Study aim

Our study aim was to explore young people’s perspectives on the factors affecting health in their local area and their perceptions of health inequalities. This paper presents key findings relating to young people’s perceptions of a North-South health divide and its drivers in England, UK.

## Methods

### Overview

We worked with 42 young people (aged 13–21) from six youth groups based in the North East, South Yorkshire and London. With each group, we carried out three interconnected focus group discussions between February and June 2021, during the Covid-19 pandemic. Fifteen of the discussions were carried out online due to social distancing restrictions. However, with one group in the North East, we carried out face-to-face discussions when restrictions had eased as the youth group did not have the necessary technology to facilitate virtual data generation. The University of Sheffield granted ethical approval for the study [Reference: 037145].

Below we provide a succinct outline of our project’s methodological approach. More detailed descriptions of our methodology, as well as reflections on and ethical considerations of our approaches, are presented in published work [24, 30].

### Sampling and recruitment

We adopted a purposive sampling strategy with the aim of exploring a relevant range of perspectives [31]. Appreciating the interplay or intersections between privilege and disadvantage, and opportunity and constraints [32], while our study focused on socioeconomic inequality, we aimed to recruit a diverse sample of young people in terms of: socioeconomic background, ethnicity, gender and urban/rural (including coastal) locations. However, recruiting during a pandemic proved challenging and we took the pragmatic decision to work with youth groups with whom we had pre-existing relationships, all of which were within areas in the most deprived quintile of the English Index of Multiple Deprivation (IMD). Working with our project partners, we were able to achieve diversity in relation to our sampling criteria across these groups (see Table 1).

**Table 1:** Participant demographics (reproduced from Fairbrother, H. et al. (2022) [24])

Sample	Number of Participants	Age	Gender	Ethnicity	Deprivation Position
Overall	42	Age range: 13–21 Average age: 16.7	18 Female 19 Male 2 Non-binary 2 Trans Male 1 Gender-Fluid	30 White British 6 Asian/Asian British 3 Black/Black British 2 Mixed/Multiple ethnic group 1 Chinese	Average participant position = 8096 (Quintile 2)
South Yorkshire 1 (SY1) (urban)	6	Age range: 15–17 Average age: 15.5	3 Female 2 Male 1 Gender-Fluid	6 White British	Average participant position = 8009 (Quintile 2)
South Yorkshire 2 (SY2) (urban)	8	Age range: 13–17 Average age: 15.1	3 Female 5 Male	8 White British	Average participant position = 9414 (Quintile 2)
North East 1 (NE1) (rural, coastal)	7	Age range: 15–17 Average age: 15.8	2 Female 1 Male 2 Non-binary 2 Trans Male	7 White British	Average participant position = 15,004 (Quintile 3)
North East 2 (NE2) (rural, coastal)	8	Age range: 13–20 Average age: 15.75	8 Male	8 White British	Average participant position = 1351 (Quintile 1)
London 1 (L1) (urban)	10	Age range: 16–21 Average age: 18.7	8 Female 2 Male	1 White British 5 Asian/Asian British 3 Black/Black British 1 Mixed/Multiple ethnic group	Average participant position = 7065 (Quintile 2)
London 2 (L2) (urban)	3	Age range: all aged 20 Average age: 20	2 Female 1 Male	1 Asian/Asian British 1 Mixed/Multiple ethnic group 1 Chinese	Average participant position = 7734 (Quintile 2)

Youth leaders shared a project information video with their members which provided an overview of the research and what participation would involve. Following this, researchers attended youth groups’ sessions to talk through the study. Young people who expressed an interest in participating were provided with an information sheet and, for participants under the age of 16, an opt-in consent form for parents/guardians. All participants provided written consent and filled out a short demographic questionnaire, including postcode, which we used to generate an overall deprivation rank measure (average position out of the 32,844 small geographical areas (lower layer super output areas (LSOAs)) in England, with closer to 1 being more deprived). Although all youth groups were based in the most deprived quintile (quintile one), the average participant positions were between quintiles one and three.

### Data generation

We partnered with youth organisations not involved in the research to develop and refine our focus group discussion plans which were guided by our aim to create safe, supportive contexts to discuss the potentially stigmatising topics of health and inequality. All of our sessions were piloted with young people through youth organisations in the North East and South Yorkshire. Key strategies included: the use of focus groups (to provide an opportunity for mutual support from fellow known participants) with youth workers present (a trusted adult), careful use of language (avoiding stigmatising terminology), open question framing so that participants did not feel pressured into sharing their own personal experiences, and ensuring at least a week between consecutive focus group discussions (to allow time to reflect on and discuss the sessions with peers and youth workers) (see Woodrow et al. 2022b [30]). In the first focus group we engaged in participatory concept mapping [33] to explore young people’s perspectives on key factors affecting their health in their local area. Participants identified and discussed factors that make it easier or harder to be healthy where they live and explored ways in which those factors are linked. The process of constructing the maps enabled young people to articulate, visualise and draw links between the complex ways in which multiple factors interrelate and interact to influence health. In the second session we explored young people’s perceptions of health inequalities using prompts from contemporary newspaper headlines. We presented a variety of topics for discussion (Covid-19, mental health, healthy eating and diet, and physical activity/sport (see Supplementary File 1 [reproduced from Fairbrother, H., Woodrow, N., Crowder, M., Holding, E., Griffin, N., Er, V., Dodd-Reynolds, C., Egan, M., Scott, S. and Summerbell, C., 2022. ‘It All Kind of Links Really’: Young People’s Perspectives on the Relationship between Socioeconomic Circumstances and Health. International journal of environmental research and public health, 19(6), p.3679] [24])). Each topic had several news headlines, with the same topics and headlines offered to all groups. One of the headlines was: ‘Coronavirus: Northern England ‘worst hit’ by pandemic’ [34] (See supplementary File 2). In the third session we discussed priorities for change to improve health within the local area.

### Data analysis

We employed thematic analysis, guided by Braun and Clarke’s (2006) thematic analysis framework [35]. We took an interpretive approach acknowledging the active role of researchers in making sense of data and generating themes. Our approach included familiarising ourselves with a selection of different transcripts (from different youth groups and for different sessions) as well as drawing reference from key concepts which had informed our study and our focus group topic guides. A coding framework was developed and refined through discussion among the research team. Data management was facilitated through the use of NVivo-12. The analysis for this paper was carried out by the lead authors (HF and NW). When reporting our findings, to minimise any potential for identification, we have taken the decision to prioritise participant confidentiality. Where we use verbatim extracts, we provide only the field site location (NE = North East, SY = South Yorkshire, L = London) and focus group session (e.g. L 1.2 = London Group 1, session 2), to protect participant confidentiality whilst maintaining geographic context. This was seen as important for ensuring participation during our study design and recruitment.

## Findings

Young people from all geographical areas demonstrated an awareness of a North-South divide in England. They described the divide in terms of stark inequalities in local economies and employment opportunities and, ultimately, health and wellbeing. They also identified how the Covid-19 pandemic had both highlighted and intensified these inequalities. Discussion of a North-South divide was more common among young people living in the North of England, and particularly prominent in the narratives of young people living in the North East.

The first and second sections below provide detail on young people’s perspectives on inequalities in the structural factors operating in the North vs. the South of England. The third section focuses on how such structural factors and disparities in employment were recognised as leading to inequalities in health and wellbeing between the North and the South. The final section then discusses young people’s perspectives on the impact of the Covid-19 pandemic on the North-South divide and how this impacted health, and brings together perceptions about the relationship between the structural differences and health impacts.

### Longstanding inequalities in local economies and employment opportunities between the North and the South of England

Young people described clear contrasts between the North and the South of England in relation to local economies and employment opportunities. Their narratives were rooted in a historical perspective, focussing on different responses to and impacts of deindustrialisation in the North and the South. Young people described how the effects of deindustrialisation upon local labour markets had had a particularly devastating impact on Northern areas with fewer jobs now available and lower-paid work:

*It depends on where you were born*,* essentially*,* because here in the North East there’s not really many job opportunities compared to in the South.* (NE 1.2)

‘[There’s] *not as many jobs as before*,* whole villages were employed and now people in*,* who were in*,* manual jobs are working for less money. (SY2.1)*

The emphasis on ‘whole villages’ in the second quote captures the participants’ focus on place-based understandings of inequalities. They vividly articulated how different areas had been hit by deindustrialisation. Young people described the post-industrial landscape of the North as dominated by hospitality and service sector employment. They contrasted this with the South, and particularly London, which they perceived to be centred around more knowledge-based, highly skilled (and better paid) employment opportunities. In summarising a group discussion, one participant noted:

*Yeah*,* we talked a lot about how the economy of the area and what jobs and the North-South divide can really affect a lot of these things. We made a point that*,* especially in response to deindustrialisation*,* different cities are funded in different ways based on the amount of support that they already had from the government and the amount of money that they already had. So some cities*,* especially in the North*,* in response to the deindustrialisation*,* replaced those jobs with things that aren’t*,* as like*,* sustainable. So things like call centres*,* the service industry*,* so things like working in retail*,* and obviously in the South*,* more like knowledge-based industries.* (NE 1.2)

The phrase ‘can really affect a lot of things’ at the start of this quote epitomises how, for young people, these contrasting post-industrial landscapes had far-reaching, myriad repercussions. For example, young people consistently emphasised the limited employment opportunities in the North, with this contributing to a vicious cycle of poverty in which unemployment and low-paid jobs led to financial insecurity: *Obviously*,* we know the North tends to be poorer and [there] tends to be more people living in poverty*’ (NE 1.2).

While young people articulated differences and inequalities within their local areas (with larger cities such as London and Manchester generally seen to receive and benefit from better, or at least greater employment opportunities), and whilst they acknowledged that the picture was not uniformly positive for the South, they emphasised a clear, overarching contrast between the North and the South:

*[…] I feel like as*,* like*,* as*,* like*,* as a whole that South of England has just got more investment than North of England*,* I know there’s some areas of South England which are*,* kind of*,* like*,* which aren’t*,* like*,* doing well in terms of economy or like money*,* for example*,* like Jaywick (Essex) and stuff like that. But*,* yeah*,* I just think*,* especially*,* like*,* more*,* like*,* companies which are from South England and stuff like that they*,* they*,* they’re more supported than the ones up North.* (L2.1)

### Challenges of moving to secure opportunities in the capital - nuanced perspectives beyond a North-South dualism

Young people in the North East and South Yorkshire groups consistently associated the South, and London in particular, with better job opportunities. A running thread in their discussions was that to go far in life, you need to go far away from the North. However, while young people acknowledged the potential to move away from the North in search of better job opportunities, they pointed out that in reality this was not viable due, in large part, to the prohibitive cost of housing further South, particularly in London:

*If you’re from a low income family*,* and you haven’t got a good job*,* but you want to go somewhere to get a good job*,* no matter how much we save up*,* if we go somewhere to get a better job we’ll waste all our money just trying to rent stuff cos it’s more expensive in other places*,* and we’ll have to find a house and stuff […] we could save all our pocket money and yeah we could go down London and waste it all in a month renting and stuff like that.* (NE 2.1)

The London youth groups emphasised the exorbitant cost of housing in the capital too but also highlighted the lack of green space and high levels of air pollution (both detrimental to health) in Central London in particular:

*But I think like you can kind of get stuck in a cycle as well*,* if you’re living in Central London and you have to live there because you’re close by to work […] you can get stuck in a cycle because it’s so expensive in Central London so then because it’s so expensive you’re spending your money on other stuff you won’t be able to afford to move out to a wealthier area*,* where there’s like potentially*,* I don’t know, more green space and less air pollution* (L2.1).

Londoners also contrasted the lack of green space and overcrowding of Central London with boroughs further out where wealthier people chose to live. They highlighted the preponderance of service sector employment in Central London, which they perceived to serve the needs of wealthier people further out of London. In this way, their narrative challenged the perception that London is uniformly dominated by highly skilled, knowledge-based job opportunities. They highlighted a perception that high concentrations of low-skill employment in central London goes hand in hand with overcrowded living conditions: ‘*There’s more overcrowding because … that’s where the labour is concentrated. Like services*,* like shops*,* like working in Tesco*,* Lidl’s or like working as cleaners […]’* (L2.1). In this way, young people in London recognised the challenges of living in the capital and highlighted that it was not always a positive, health promoting experience and so did not always represent a positive contrast to the North. Further, young people in all areas perceived the cost of housing (particularly in the South and in desirable areas of London) to be a real barrier to geographical, social and economic mobility.

### Disparities in employment opportunities lead to inequalities in health and wellbeing between the North and the South

In line with the assertion that the North-South divide ‘can really affect a lot of things’ (NE 1.2), young people described different ways in which disparities in employment opportunities ultimately lead to inequalities in health and wellbeing between the North and the South. They associated the contemporary employment landscape in the North with negative physical and mental health impacts:

*So if you’re working in a physical job that’s taking a toll on you*,* the way that your body reacts to that is obviously you’re at higher risk to things like heart disease and that sort of thing. But that’s visible and you can see that*,* whereas a lot of things to do with like the service industry*,* it can lead to things like anxiety and depression*,* which aren’t as visible and you might not get help as quickly because either you don’t notice it or other people don’t notice it in you*. (NE1.2)

The participant’s emphasis on the importance of the potentially hidden mental health impacts of service sector work (which the Northern participants saw as dominating their local labour markets), compared with the ‘obvious’ and ‘visible’ physical health impacts ‘that you can see’ of physical jobs, is important. Further, while factories were described as potentially offering a better income than service sectors this had to be weighed up against the ‘high-risk’ nature of factory work: ‘*You’re going to get paid more working in a factory than you are working in a call centre. You’re more likely to die so you do make less money [in call centres] because of that high-risk environment bit*’ (NE 1.2). In this way, young people perceived a ‘choice’ between high-risk and better pay or low-risk (at least in terms of immediate risk to physical health), low-pay work.

As well as direct occupational health risks and impacts, young people described how the employment landscape in the North could lead to negative health practices. For example, one group of young people associated high levels of substance misuse within their local area with high levels of unemployment and poorly paid work. In this extract, the participants allude to a pernicious ‘ripple effect’ through the community as local industry recedes and people are left with fewer job opportunities and lower incomes:

*Participant 1: I would say that Northern England would be poorer because they used to be mining towns so are more likely to have less money now that they are closed. I think people are a product of their circumstances when it comes to substance abuse.[…] not as many jobs as before*,* whole villages were employed and now people in*,* who were in manual jobs are working for less money.*

*Participant 2: almost like a domino effect I think one area falls and then another follows suit* (SY.1.2).

Later in the conversation, the participant articulates in more detail what they mean by ‘people are a product of their circumstances when it comes to substance abuse’ as they describe how unemployment and low-paid work have a detrimental impact on self-esteem ‘*which could lead to substance abus*e’ (SY1.2). Here then there is a real sense of the interweaving of people’s health practices and their social and economic context. Young people’s narratives underscore how difficult it is to disentangle people and health from place.

As well as pushing people towards negative health practices, young people also described how local economies and working conditions in the North were making it practically harder for people to engage in positive health practices, like cooking for a family:

*We did talk a bit about how people in the North*,* the sort of jobs that we have*,* it’s less likely that you’ll be able to work from home. So if you are working from home – which predominantly*,* especially if you’re in the South because a lot of the economies*,* they’re very knowledge-based – you can afford to do that sort of thing from home. And you might only need to go into the office every couple of days. So they have certainly got more time and more time that they can dedicate to something like cooking. Whereas if you are still having to go to work*,* especially with everything that’s going on*,* I can imagine that must be really stressful. You’re probably not going to dedicate as much time to cooking and looking after your family and that sort of thing.* (NE1.2)

So better access to employment opportunities in the knowledge-based economies of the South was perceived to afford greater flexibility in working practices and ultimately more time to dedicate to health promoting practices.

A sense of fatalism permeated young people’s accounts of a North-South divide as they described an interweaving of regional and intergenerational inequalities over time. One participant poignantly articulated the sense of inequity and feeling of hopelessness: *‘It’s actually unfair. The facts are right there in front of your eyes*,* because if you’re born quite a poor person*,* then most people would expect you to stay poor and vulnerable to a lot of diseases’ (NE 2.2)*. The phrase *‘the facts are there in front of your eyes’* highlights young people’s assertion that these differences are not up for debate - they are clearly apparent and visible to everyone. Children were described as victims of unemployment, parental poor mental health and poverty:

*The kids have no choice in whether they’ve ended up in poverty or not*,* because it’s the parents who pay for everything. It’s the parents who either do or don’t have the job. It’s the parents who do or don’t have the mental problem that has caused them to go into poverty.* (NE 1.2)

The quote hints at the inextricable links and complex, two-way relationships between (un)employment and mental health. There is a blurring of the cause and consequence. Again the phrase ‘the kids have no choice’ echoes the assertion that ‘people are a product of their circumstances’. In this way, young people were acutely aware of both direct and indirect health and wellbeing impacts of the challenging employment landscape in the North.

### The Covid-19 pandemic exposed and amplified the North-South divide

The Covid-19 pandemic was perceived to have exacerbated the North-South divide in employment opportunities. Describing the local employment landscape pre-Covid as ‘not very good at all’, one participant described how the Covid pandemic had compounded unemployment and how the social security system was insufficient to support the people affected by this:

*Like because of Covid*,* places had had to shut down*,* meaning they won’t have any income. And the services that help people*,* like Universal Credit*,* they’re losing a lot of money as well so they can’t really help people.* (SY1.2)

Young people also talked about differential exposure to the coronavirus due to the differences in local labour markets referred to earlier. Greater opportunities to work from home were perceived to be protective for people in the South (though it is also important to recognise the London participants’ emphasis on the preponderance of service sector employment in Central London compared to wealthier areas further out):

*I think that the type of job as well that you’re doing*,* because I know a lot of the ones in the South are like knowledge-based*,* so you can work from home pretty easily*,* but I know a lot more people in the North – just particularly looking at the type of jobs we have*,* being like in shops and in – well*,* call centres*,* it’s not really something that you can do from home as easily. So it’s meaning that they’re more exposed to it.* (NE 1.2)

Young people across the groups thought that the pandemic had served to highlight long-standing disparities in government investment between the North and the South. One of the London participants eloquently explained how the Conservative government had focused on supporting the South and neglected the needs of the North:

*I know that in North England people are not as wealthy as the South England*,* kind of thing. Because obviously*,* like the government*,* well*,* over the recent years the government’s basically just been focusing on the South of England because*,* yeah*,* that’s where the capital is and it’s a bit more*,* the economy in the South of England’s a lot better than the North. So I guess*,* the pandemic has highlighted the fact that they’ve been*,* the government has*,* kind of*,* been putting the north on the side and just*,* like*,* yeah*,* not paying attention to their needs as much*. (L2.1)

Better funding in recent years was perceived to have resulted in businesses in the South being more resilient to shocks like the pandemic. One of the young people from the North East thought that this was because politicians in central government ‘care about where they live’ (NE 1.2). The pandemic was perceived as exposing and exacerbating inequalities in employment opportunities and working conditions between the North and the South and highlighting disparities in funding for business and health services, motivated by self-serving concerns of politicians.

## Discussion

### Summary of findings

Young people in our study described clear contrasts between the North and the South of England in relation to local economies and employment opportunities. Their narratives were rooted in a historical perspective, focussing on different responses to and impacts of deindustrialisation in the North and the South. Moving away from the North, in search of better job opportunities, was perceived to be difficult for many young people. The cost of housing (particularly in the South and desirable areas of London) was perceived as a real barrier to geographical and social and economic mobility. Young people associated the contemporary employment landscape in the North with precarity, poor pay and negative physical and mental health and wellbeing impacts. They described both direct occupational health risks and also differences in health practices related to poorly paid work, unemployment and a lack of autonomy and flexibility for workers. They also highlighted how the Covid-19 pandemic had highlighted the extent of and exacerbated the North-South divide, underscored disparities in investment for business and exposed the vested interests of the London-based governing elite. Throughout their narratives, young people demonstrated that they were acutely aware of, and could articulate in detail, the impact of national decision making, challenging perceptions of a ‘politically disengaged’ generation.

### The long shadow of deindustrialisation for communities in the North

Our study highlights that young people are acutely aware of deep-rooted and longstanding regional disparities in the building blocks of good health [9, 10]. They continue to experience the fallout of deindustrialisation in the North and their narratives echo the extensive evidence base highlighting how formerly thriving industrial areas of England and the UK are now characterised by persistent, intergenerational deprivation [36]. The discussions of young people from the North East in particular echo work by Shildrick et al., (2012), from over a decade ago, which explored poverty and insecurity among men and women both young and old in Middlesbrough, the main town of Teesside in North East England [37]. Just like our participants, the participants in Shildrick et al.’s (2012) study highlighted a lack of available jobs in the local market and a preponderance of ‘poor quality jobs that trapped them in long-term insecurity and poverty’ [37 p.3]. The narratives of young people in our study also resonate with work by Mackenzie et al. (2017) with local communities in two deindustrialised areas in Scotland [38]. Like our participants, people in Mackenzie et al.’s (2017) study had ‘highly integrated views of health, including vivid articulations of links between politics, policies, deindustrialisation damage to community fabric and impacts on health’ [38 p.231]. However, young people’s focus on structural inequality in our study stands in sharp contrast to recent survey work by IPSOS Mori which found that the large majority of respondents thought that the UK was meritocratic with an ‘unwavering belief [...] that while structural factors play a role in people’s experiences of inequality, it was ultimately up to the individual to improve their life chances’ [39 p.9]. However, this perception was more common among older than younger participants in the survey.

Young people’s focus on differences in employment opportunities between the North and the South and particularly their focus on London as the centre of the ‘knowledge economy’ in our study coheres with recent analysis from the Fabian Society (2023) which demonstrates how economic underperformance in regions outside London and the South East combine to make the UK ‘the most regionally unequal developed country’ [40 p.5]. Our findings show how these issues can be experienced, perceived and understood by young people. This economic underperformance is evident in regional disparities in both productivity and household income and is getting worse. For example, London and the South East have secured 45% of net job growth in England since 2010 while the North East has secured only 2%. Jobs are becoming increasingly concentrated in London and recent research demonstrates that London is increasingly attractive to investors - with London boroughs taking nine of the top ten places for economic competitiveness out of 362 local areas [41]. The area fatalism evident in young people’s accounts - their articulation of the pernicious ripple effect through the community as local industry recedes and people are left with fewer job opportunities and lower incomes - should serve as a stark warning to policy which simply focuses on raising young people’s aspirations [42]. For the young people in our study, like the participants in Shildrick et al.’s (2012) study, many of the jobs available to them were perceived to ‘neither relieve poverty nor provide pathways up and away from it’ [37 p.194] - highlighting a depressing continuity in young people’s labour market experience [43]. Their perceptions reflect the reality of regional economic inequality with patterns of poorly paid, temporary jobs and unemployment still ‘a permanent feature of life for economically marginalised groups’ [37 p.5). The emphasis on the prohibitive cost of housing in London in the accounts of young people across all of our groups also challenges a focus on raising aspirations of young people living in contexts of deprivation [42], without adequate means to fulfil them (see also Gbohoui et al., 2019 [1]). Whereas in recent polling for IPSOS Mori respondents perceived the higher costs of housing in London to be acceptable due to higher salaries in the capital [44], the young people in our study highlighted how for (young) people starting out or trying to secure employment in the capital, housing costs represent a real stumbling block.

### Health and wellbeing impacts of poor employment opportunities

Our study supports previous research exploring public understandings of the fundamental causes of health inequalities, and echoes Watt et al.’s (2022) assertion that the North-South [health] divide reflects regional imbalances in ‘average incomes, wealth, economic opportunity and educational attainment’, and ‘speaks to the strong relationships between inequalities in the wider determinants of health and inequalities in diagnosed ill health’ [11 para.5]. In particular, our study highlights a nuanced understanding of the inextricable link between employment and health and wellbeing. It reflects Shildrick et al’. s (2012) finding from work in the North East that available work was ‘typically physically and mentally demanding and yet poorly valued in terms of remuneration and status’ [37 p.7]. It also coheres with recent research with young people in the north of the UK (Leeds and Glasgow) exploring policy priorities for reducing inequalities in health [45]. Here, ‘links were frequently made between employment and mental health in ways that align with reviews of research evidence around employment as a driver of mental wellbeing’ [45 p.10].

Young people’s discussions about the health and wellbeing impact of unemployment echoed those of Mackenzie et al.’s (2017) participants who foregrounded the psychosocial impact of unemployment, particularly in terms of loss of self-esteem [38] (see also Minh et al., 2020 [46]). Our participants’ discussions of the ways in which unemployment led to negative health practices like substance misuse also echoes wider evidence demonstrating a higher prevalence of risk behaviours like smoking and alcohol consumption amongst the unemployed [47]. Similarly, the emphasis on unsocial hours, lack of flexible working and the ways in which this limited opportunities for positive health practices like cooking for a family supports Strazdin et al.’s (2016) argument for considering time a social determinant of health [48]. In this way, young people’s narratives highlight a movement away from previous issues faced by working class people (chronic health issues from manual labour) to less tangible issues resulting from uncertain, insecure, low paid and temporary service sector work [49] which they perceived to dominate in the North. Such forms of precarious employment are a national issue in the UK, and not exclusively a Northern issue, but young people perceived them as important in their understanding of regional inequality. Further, while young people’s narratives foregrounded psychosocial pathways linking poor employment opportunities to health and wellbeing, their narratives also highlighted nuanced understandings of the interweaving of material, psychosocial and behavioural mechanisms [24].

### A politically driven neglect of the North

Young people’s emphasis on the role of central government in creating and perpetuating a North-South divide echoes recent survey-based research by IPSOS Mori in which nearly half of respondents (48%) thought politicians ‘paid more attention to some areas than others’ [39 p.5]. It also echoes qualitative work with communities in deindustrialised areas of Scotland, where participants highlighted a politically driven failure to invest in Scotland with policies reflecting the ‘minority economic interests’ of the ‘powerful elite’ [38 p.238]. Our young people’s focus on the vested interests of the government reflects recent research by Fergie et al., (2023) in which young people articulated an awareness of how ‘existing democratic arrangements perpetuated inequalities in power, in ways that [...] predicated health inequalities’ [45 p.7]. Indeed, their focus on a lack of investment in the North is supported by recent work from the Institute for Public Policy Research, where levels of public investment in London and the South East are compared with the North [50]. Here the authors highlight that a funding gap between the North and the South ‘has persisted for decades, and has actually increased since the Northern Powerhouse agenda was first announced in 2014/15’ [50 p.14]. For example, they highlight that: ‘In the five years to 2019/20, London received £12,148 per person, which is over £4,000 more than the £8,125 invested per person in the North’ [50 p.14]. Young people clearly recognise the importance of these policy decisions to their own life chances and subsequent health outcomes.

In our study, the young people from groups in the North, and particularly the North East spoke more frequently about a North-South divide. This echoes recent survey work showing that people in the North were more likely than those in the South to ‘express concern’ about a lack of attention, money and resources for the North from government politicians [39 p.5]. It is perhaps unsurprising that our participants from the North East were the most vocal about regional inequalities as the North East consistently ‘tops the charts’ for both poverty and poor health [22], highlighting how their lived experiences shaped their perceptions. Similarly, young people from London spoke more about the stark inequalities they witnessed within the London boroughs where they lived.

### Study limitations and strengths

A limitation of this study is that our sample was confined to young people living in contexts of deprivation and does not, therefore, engage with the ways in which people from contrasting social positions experience and perceive health (and other) inequalities [51]. Additionally, while our London groups were ethnically diverse, all participants in the groups we worked with in the North East and South Yorkshire were White British. Further, we acknowledge that by not providing individual participant demographic information for quotes, motivated by a commitment to confidentiality, we cannot explore how understandings relate to individual characteristics (e.g. gender or socioeconomic position) or changes in understanding for individuals over the course of the three data generation sessions. We also recognise that many youth organisations discuss topics like health and inequality and therefore the young people we worked with may have had more developed thoughts about these subjects and be more ‘socially engaged’ than young people who are not part of youth groups. Further, since young people’s narratives regarding a North-South health divide arose in the context of a wider discussion of place-based health inequalities, our findings do not provide a clear, geographical definition of North and South. Indeed, while there is a popular discourse around the North-South divide, this may not necessarily strictly adhere to a clearly specified geographical boundary.

Generating data with young people over the course of three interlinked sessions is a key strength of our study. This helped in building trust and rapport and creating a safe and supportive space in which to challenge each other. It also provided opportunities to develop and refine understandings and to revisit early discussions as a form of sense-checking and reflection. Carrying out the focus groups during the Covid-19 pandemic also created a unique opportunity to discuss inequalities and highlights the importance of recognising the context in which research is carried out [44]. Our study affords important insights into how young people experience and understand geographically patterned socioeconomic and health inequalities in the context of their lives. In doing so, it contributes to developing our understanding of the causal pathways, processes and relationships through which social and economic inequalities create health inequalities [52]. In particular, our participants’ focus on disparities in employment opportunities and the negative health and wellbeing impacts of low-paid and low-skilled work helps to address the dearth of studies exploring public perceptions of the role of occupation in health inequality [53]. The study underscores quantitative analyses highlighting longstanding and growing regional disparities between the North and South in relation to incomes, job creation and unemployment.

### Priorities for future research

Future research should ensure that the perspectives of young people living in contexts of socioeconomic advantage are explored and compared and contrasted with the perspectives of young people living with deprivation. Our study did not set out to specifically discuss the North-South divide so there is potential for more focussed work here, including exploring how different axes of inequality intersect to shape opportunities for good health and the interrelated mechanisms involved [54]. Developing a greater understanding of how key social policies play out in the context of people’s everyday lives and their relevance to health and wellbeing is also important [55]. Further, our work supports recent calls for researchers to ‘better synthesise and systematise available evidence to address policy questions’ and ‘work to understand the political landscape [...] to build advocacy coalitions’ [56 p.7]. By clearly articulating the problems and systematically synthesising the evidence we can demonstrate that we already have a sound understanding of the fundamental causes of health inequalities and the policy actions that can reduce them [52].

### Policy and practice implications

Our study underscores the need for place-based solutions for inequalities [9, 10, 57]. It highlights the potential of working more closely with young people in deliberative policy analysis [58] and of prioritising young people’s voices and experiences [9]. For example, it could be fruitful to draw together young people to explore their ideas for policies relevant to reducing the North-South divide in health. In particular, opportunities for young people from the North to be more involved in policy development should be increased. Further, work bringing *together* young people from *different* regions to unpack and better understand how place affects inequality would be valuable. Overall, our study foregrounds the importance of investment in the North to tackle inequalities in employment opportunities and income [59]. In this respect, we can look to learn from our European neighbours like Germany and France whose rates of local and regional economic spending far outweigh the UK’s [40]. Linked to this, young people’s emphasis on an out-of-touch London-based governing elite (which all groups agreed on and which impacts both the North-South issue and inequalities within regions) also lends support for calls to devolve economic and fiscal power to regional and local government. The introduction of new mayoral combined authorities is showing promising signs here [59] but again we can learn from countries like France and Germany where regional governance is much more autonomous than it is in the UK [39, 60].

## Conclusions

Young people’s narratives demonstrate their awareness of the interplay between spatial, social and health inequalities - they highlight experiential understandings of how opportunities to enjoy good health are inextricably linked to where people are born, live and work. However, while inequalities between the North and the South are entrenched and increasing, they are not inevitable [60]. Structural inequalities represent a ‘design fault in our systems and institutions’ and young people’s emphasis on the role of government in creating and perpetuating the North-South divide highlights that inequalities are ‘there by design’ but also that we can ‘design them out’ [61].

### Electronic supplementary material

Below is the link to the electronic supplementary material.

Supplementary Material 1

Supplementary Material 2

## Data Availability

The datasets generated and analysed during the current study are not publicly available due to privacy reasons but are available from the corresponding author on reasonable request.
