# Supplementary material for: ‘It depends on where you were born…here in the North East, there’s not really many job opportunities compared to in the South’: young people’s perspectives on a North-South health divide and its drivers in England, UK
Source: BMC Public Health. 2024 Jul 29;24:2018. doi: 10.1186/s12889-024-19537-z (PMC11285465; doi:10.1186/s12889-024-19537-z)
Supplement: Supplementary file 2 — Supplementary Material 2 [file 12889_2024_19537_MOESM2_ESM.pdf]

1. COVID-19
2. Mental Health
3. COVID-19 and Mental Health
4. Healthy Eating
5. Free School Meals
6. Physical Health (exercise)

COVID and Health Inequalities

The Guardian

Covid-19 has exposed the reality of Britain: poverty, insecurity and inequality

BBC | Home | News | More

NEWS

Home | Coronavirus | UK | World | Business | Politics | Tech | Science | Health | Family & Education | Entertainment & Arts

England | Local News | Regions

Coronavirus: Northern England 'worst hit' by pandemic

11 November

The Guardian

How do we 'build back better' after coronavirus? Close the income gap

Mental Health and Health Inequalities

Morning Star for Peace and Socialism

Children’s mental ill-health: a symptom of poverty and inequality

Rather than individualising kids’ problems, they should be looked at in context, says STEVEN WALKER

Mail Online

Three quarters of young people 'manage their mental health problems themselves' because they can't get help, charity warns

- They struggled to get help online and didn't feel comfortable confiding in others
- Pressures at school, social media and appearance all factors in causing issues
- It has been shown that mental health plunges if youngsters don't get initial help

The Guardian

Young people’s mental health is a ‘worsening crisis’. Action is needed

Covid-19 and Mental Health

MailOnline

One in six children has a mental health problem and cases have rocketed by 50% during coronavirus pandemic and lockdowns, new report shows

- NHS study revealed one in six youngsters now had a condition such as anxiety
- Experts blamed 'distressing' figures on school closure and worries over Covid
- A quarter of young women aged 17 to 22 identified 'probable mental disorder'

BBC | Home | News | More

NEWS

Home | Coronavirus | UK | World | Business | Politics | Tech | Science | Health | Family & Education | Entertainment & Arts

UK | England | N. Ireland | Scotland | Alba | Wales | Cymru | Local N

Coronavirus: Teens' anxiety levels dropped during pandemic, study finds

24 August

Healthy Eating and Health Inequalities

MailOnline

Study reveals parents' poor eating habits are to blame for childhood obesity, NOT fast food

- A study conducted at the University of North Carolina at Chapel Hill found that poor eating habits linked to obesity are learned in children's homes
- Fast food does contribute to obesity as does diets low in vegetable and high in preservatives served at home and at schools
- Some parents don't take the time to cook healthy meals for their children, and this leads to poor dietary decisions later in life

The Guardian

Children in poor areas exposed to five times as many fast food takeaways

The Guardian

Four million UK children too poor to have a healthy diet, study finds

Free School Meals

The Guardian

Huge growth in free school meals urged to tackle food poverty crisis

METRO

Tory MP sparks fury after suggesting free school meals cash 'goes to crack dens'

Socialist Worker

Blame Tories not the poor for obesity and hunger

The rolling out of new measures to tackle obesity shames poor people and will not tackle a crisis that the Tories made worse, says Sarah Bates

Physical Health (exercise)

BBC | Home | News | More

NEWS

'Global epidemic' of childhood inactivity

BBC | Home | News | More

NEWS

Poor children lose out on exercise, research suggests
